# First person – Bishal Basak

**DOI:** 10.1242/bio.058657

**Published:** 2021-03-18

## Abstract

First Person is a series of interviews with the first authors of a selection of papers published in Biology Open, helping early-career researchers promote themselves alongside their papers. Bishal Basak is first author on ‘Interdomain interactions regulate the localization of a lipid transfer protein at ER-PM contact sites’, published in BiO. Bishal is a PhD student in the lab of Professor Raghu Padinjat at National Center for Biological Sciences, Rajiv Gandhi Nagar, Kodigehalli, Bengaluru, Karnataka, India, investigating non-vesicular trafficking of lipids at interorganeller contact sites regulate cellular physiology.


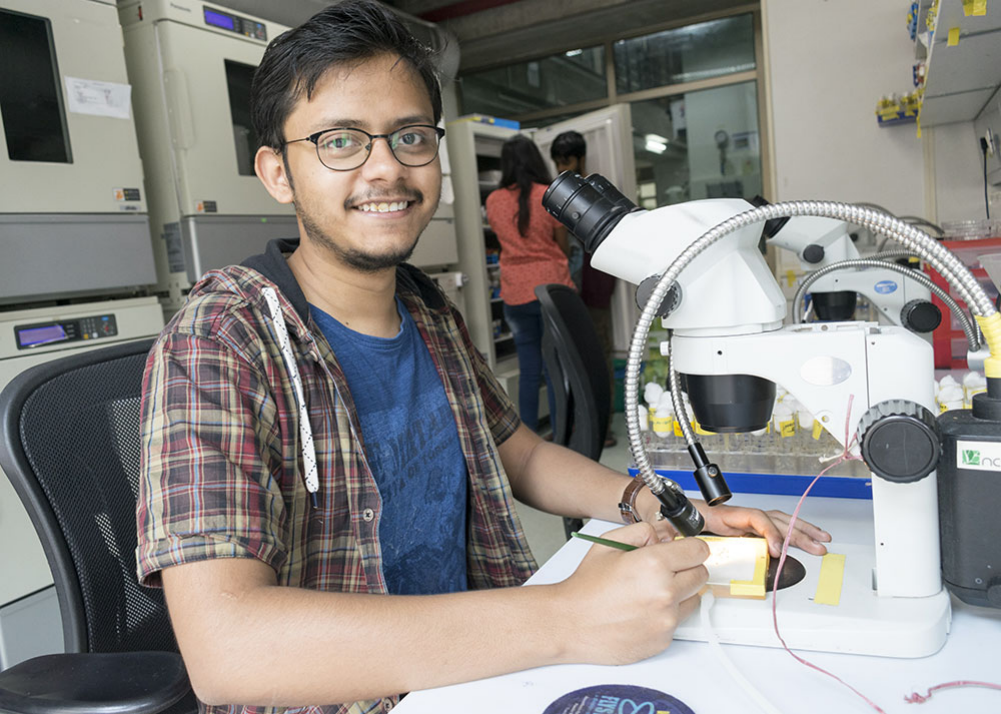


**Bishal Basak**

**What is your scientific background and the general focus of your lab?**

I joined Prof. Padinjat's lab as an Integrated MSc-PhD student soon after receiving my Bachelor's degree in Microbiology from St. Xavier's College, Kolkata, India.

I have always been intrigued with the concept of how cells communicate using simple molecules like lipids, proteins and other effectors. Such communication is essential to support physiology starting from a unicellular bacterium to the most complex eukaryotic organism. Prof. Padinjat's lab looks at the role of a specific group of lipids termed phosphoinositides in maintaining membrane trafficking, sub-cellular organization, in addition to understanding how they regulate human brain development and function. The lab provided me with an ideal platform to study how lipid mediated signalling is key to driving multiple cellular processes.

“I have always been intrigued with the concept of how cells communicate using simple molecules…”

**How would you explain the main findings of your paper to non-scientific family and friends?**

My article deciphers how the interactions of each subunit of a complex protein is essential to provide vision in insects such as the fruit fly.

**What are the potential implications of these results for your field of research?**

Since the initial discovery of Phosphatidylinositol (PI) transfer proteins (PITPs) in 1974, a long-standing question has been why some PITPs have multiple domains as opposed to few others which just harbor only a singular PI transfer domain. Our results not only address how each domain in a multidomain PITP is important to the protein per se, but also sheds light on how loss of these domains disrupt cellular homeostasis. Our detailed study on RDGB function in *Drosophila* photoreceptors is one of the few studies which deduces the importance of regulated lipid transfer at ER-PM interface from an *in vivo* cell biological perspective.

**What has surprised you the most while conducting your research?**

I have been amazed by how different domains of a protein, each with distinct role, also interact with each other to regulate neuronal structure and function.

**Figure:**
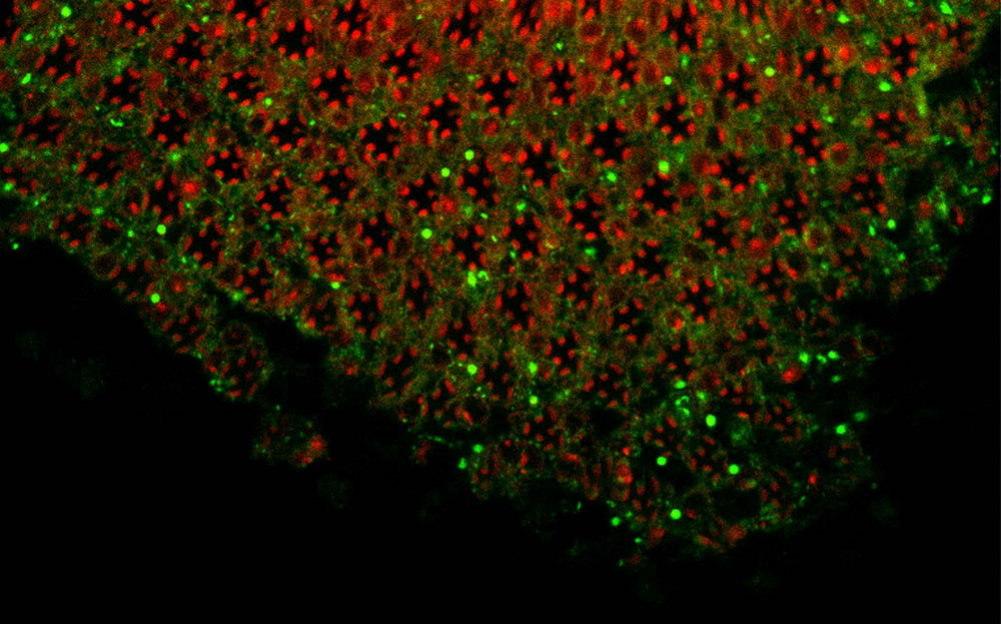
**Degeneration of photoreceptors (red) triggered by altered localization of a lipid transfer protein (green).**

**What, in your opinion, are some of the greatest achievements in your field and how has this influenced your research?**

Some of the initial discoveries which showed that blocking traditional vesicular trafficking pathways do not affect transport of certain lipids paved the path for identifying and studying non-conventional modes of trafficking within cells. Additionally, discoveries involving how photoreceptors of *Drosophila* serve as an unique and powerful model system to study the *in vivo* effects of lipid transfer at membrane contact sites, not many of which are known in the field, have been extremely influential for our study.

**What changes do you think could improve the professional lives of early-career scientists?**

I believe there should be more opportunities for early-career scientists to present their work out in the scientific community. This should include more workshops, symposiums and conferences at both national and international level dedicated for people at such career stages. In conferences there should be more student talks than faculty, online streaming/poster sessions (even after in-person conferences resume) should be made mandatory benefitting those who cannot afford the expenditures of attending an international conference. Also, symposiums dedicated particularly for career guidance will greatly impact the lives of many early-career scientists who may wish to transition from academia.

“…online streaming/poster sessions (even after in-person conferences resume) should be made mandatory…”

**What's next for you?**

I am currently working on getting another manuscript out for publication. I am also looking forward to starting working as a post-doctoral fellow soon.
